# KHSRP has oncogenic functions and regulates the expression and alternative splicing of DNA repair genes in breast cancer MDA-MB-231 cells

**DOI:** 10.1038/s41598-024-64687-0

**Published:** 2024-06-26

**Authors:** Xuelaiti Paizula, Aliya Wulaying, Dong Chen, Jianghua Ou

**Affiliations:** 1 The Affiliated Tumor Hospital of Xinjiang Medical University, Ürümqi, China; 2 The First Affiliated Hospital of Xinjiang Medical University, Ürümqi, China; 3 Innovation and Research Center, Wuhan Nissi Biotechnology Co., Ltd., Wuhan, China

**Keywords:** Breast cancer, KHSRP, RNA-seq, Alternative splicing, Cell cycle, DNA damage, Breast cancer, Mechanisms of disease

## Abstract

Breast cancer has become the most common type of cancers worldwide. Its high prevalence and malignant features are associated with various environmental factors and molecules. The KH-type splicing regulatory protein (KHSRP) participates in the development of breast cancer, while the underlying mechanisms are largely unknown. In this study, we silenced KHSRP expression in MDA-MB-231 cells by small interfering RNA (siKHSRP), and then assessed its effects on cellular features. Finally, we performed whole transcriptome sequencing (RNA-seq) experiments to explore the downstream targets of KHSRP, and validated their changed pattern using quantitative polymerase chain reaction. We found KHSRP showed higher expression level and was associated with worse prognosis in breast cancer patients. In siKHSRP samples, the proliferation, invasion, and migration abilities were significantly repressed compared with negative control (NC) samples, while the apoptosis level was increased. By investigating the RNA-seq data, we found KHSRP globally regulates the expression and alternative splicing profiles of MDA-MB-231 cells by identifying 1632 differentially expressed genes (DEGs) and 1630 HKSRP-regulated AS events (RASEs). Functional enriched analysis of DEGs demonstrated that cilium assembly and movement and extracellular matrix organization pathways were specifically enriched in up DEGs, consistent with the repressed migration and invasion abilities in siKHSRP cells. Interestingly, the cell cycle and DNA damage and repair associated pathways were enriched in both down DEGs and RASE genes, suggesting that KHSRP may modulate cell proliferation by regulating genes in these pathways. Finally, we validated the changed expression and AS patterns of genes in cell cycle and DNA damage/repair pathways. Expression levels of *BIRC5*, *CCNA2*, *CDK1*, *FEN1*, *FOXM1*, *PTTG1*, and *UHRF1* were downregulated in siKHSRP samples. The AS patterns of *PARK7*, *ERCC1, CENPX*, and *UBE2A* were also dysregulated in siKHSRP samples and confirmed PCR experiments. In summary, our study comprehensively explored the downstream targets and their functions of KHSRP in breast cancer cells, highlighting the molecular mechanisms of KHSRP on the oncogenic features of breast cancer. The identified molecular targets could be served as potential therapeutic targets for breast cancer in future.

## Introduction

Breast cancer (BC) has become the most commonly diagnosed cancer worldwide in 2020^1^, and will constantly increase to 3 million new cases and 1 million deaths in 2040 by prediction^2^. Due to the heterogeneous pathogenesis, the treatment of breast cancer should consider the underlying molecular features of patients, indicating targeted therapies could obtain better therapeutic effect^3,4^. Thus, it is important to deeply understand the molecular mechanisms of breast tumorigenesis and progression. Besides the well-studied molecules such as ERBB2, hormone receptors, and BRCA genes that are used to classify subtypes of BC and therapeutic targets, novel risk factors are identified in recent studies^5,6^. For instance, epigenetic modulators, regulating gene expression and chromatin stability, also have the ability to control estrogen level and its-targeted gene expression in BC^7^. The expression profile of long noncoding RNAs (lncRNAs) could correctly categorize BC subtypes and identify DSCAM-AS1 as a mediator for tumor progression and tamoxifen resistance^8^. Another important topic is the DNA damage and following repair, which has been tightly associated with the development and therapy of breast cancer^9,10^. In breast cancer, unrepaired DNA damage could result in malignant development of mouse luminal epithelial cells^11^. Meanwhile, the underlying mechanisms how these risk factors are regulated remain unsolved in breast cancer.

Among the regulators, RNA binding proteins (RBPs) have essential roles in post-transcriptional regulatory network^12^, and are widely associated with the dysregulated biological processes during cancer development^13^. Among the thousands of RBPs, we pay attention to the KH-type splicing regulatory protein (KHSRP), which is a multi-functional protein regulating RNA life and important cellular functions in cell differentiation and diseases^14^. As an RBPs, KHSRP regulates the fate of its targeting RNAs, including biogenesis^15^, stabilization^16^, localization^17^, alternative splicing^18^, and RNA decay^19^. It has also been reported that KHSRP involves in the tumorigenesis and progression of multiple cancers^20–22^. In breast cancer, KHSRP was up-regulated and correlated with proliferation of tumor cells and poor prognosis of patients^23^. Meanwhile, KHSRP is a downstream target of BRCA1 breast cancer cells^24^, indicating that KHSRP is an important mediator in the development of breast cancer. However, the exact functions and molecular mechanisms of KHSRP in breast cancer are still unknown.

In this study, we speculate that KHSRP may regulate the expression and alternative splicing of a large number of tumor-associated genes based on the fact that KHSRP was correlated with the development of breast cancer and poor prognosis of patients. To validate this hypothesis, the KHSRP was knocked down in human breast cancer (MDA-MB-231) cells, and the transcriptome profile affected by KHSRP was obtained by high-throughput transcriptome sequencing (RNA seq). Then, the potential targets of transcriptional and alternative splicing levels regulated by KHSRP in MDA-MB-231 cells were analyzed, and the molecular mechanism of KHSRP affecting gene expression and alternative splicing in MDA-MB-231 cells was preliminarily explored. Based on the results, we obtained a global view of KHSRP-regulated downstream targets, and understood how KHSRP promotes breast cancer progression; the identified molecular targets could be used as potential targets for breast cancer therapy in future.

## Materials and methods

### Cloning and plasmid construction

All siRNA duplexes were purchased from Gemma (Suzhou, China). Non-targeting control siRNA(siNegative) sequences were 5ʹ-UUCUCCGAACGUGUCACGUTT-3ʹ (sense). The siRNA targeting KHSRP (siKHSRP) sequences were 5ʹ-GCGAGAAUGUGAAAGCCAUTT-3ʹ (sense).

### Cell culture and transfections

MDA-MB-231 cell line (Procell, Wuhan, China) was cultured at 37 °C with 5% CO_2_ in Leibovitz's L-15 with 10% fetal bovine serum (FBS), 100 µg/mL streptomycin, 100 U/mL penicillin. The siRNA transfection into the cells was performed using Lipofectamine™ RNAiMAX transfection reagent (Invitrogen, Carlsbad, CA, USA) according to the manufacturer's protocol. We collected the transfected cells after 48h for following experiments.

### Western blot

MDA-MB-231 cells were lysed in ice-cold wash buffer (1× PBS, 0.1% SDS, 0.5% NP-40 and 0.5% sodium deoxycholate) supplemented with a protease inhibitor cocktail (Roche) for 30 min. Samples were boiled for 10 min in boiling water with 1X SDS sample buffer and separated on 10% SDS-PAGE. With TBST buffer (20 mM Tris-buffered saline and 0.1% Tween-20) containing 5% non-fat milk power for 1 h at room temperature, membranes were incubated with primary antibody: KHSRP antibody (1:1000, A9075, ABclonal) and GAPDH (1:5000, ATPA00013Rb, AtaGenix), and then with HRP-conjugated secondary antibody (anti-mouse or anti-rabbit 1:10,000) (Abcam). Then the samples were detected using the enhanced chemiluminescence (ECL) reagent (Bio-Rad, 170506).

### Cell apoptosis and proliferation experiments

We assessed the cell apoptosis and proliferation levels by flow cytometric and CCK-8 kit analysis, respectively, following the published methods^25^. The siKHSRP and NC cells were prepared with 5 μl Annexin V-APC and then mixed with 5 μl 7-AAD reagents following the instruction of Annexin V-APC/7-ADD apoptosis detection kit (40304ES60, Yeasen, Shanghai, China). Then, the cells were used for flow cytometric analysis (FACSCanto, BD, Franklin Lakes, NJ, USA) to assess apoptosis level. For proliferation, equal cells were seeded in culture plates. Then we added 10 μl CCK-8 solution and measured the optical density (OD) by Microplate Reader (ELX800, Biotek, Winooski, VT, USA) at an absorbance of 450 nm, and finally assessed the proliferation level.

### Cell migration and invasion assays

The migration and invasion assays were both performed by transwell chambers (3,422, Corning, Corning, NY, USA) with 8 μm filter. For migration, 2.5 × 10^5^ cells were added to the chambers, which were inserted into medium with 600 ul 10% FBS (10091148, Gibco, Waltham, MA, USA). For invasion, the bottom chambers were precoated with a uniform layer of Matrigel (356234, BD Biosciences, San Jose, CA, USA) and diluted for 1:8 using serum-free medium. The diluted matrigel in chambers was incubated for 1 h at 37 °C with 5% CO_2_ and removed unsolidified supernatant. Following this, 4 × 10^5^ cells were added to the inserts. The chambers were treated with the same amount of FBS as migration assay. Finally, the migrated and invaded cells were calculated under an inverted microscope (MF52-N, Mshot, Guangzhou, China) at 200× magnification.

### RNA extraction and sequencing (RNA-seq)

Total RNAs were extracted from MDA-MB-231 cells using TRIzol Reagent (NO 15596026, Invitrogen) following the canonical RNA isolation method^26^. DNA was digested by DNaseI. RNA quality and integrity were determined by examining A260/A280 with NanodropTM OneCspectrophotometer (Thermo Fisher Scientific Inc) and by 1.5% agarose gel electrophoresis, respectively. Then RNAs were quantified by Qubit3.0 with QubitTM RNA Broad Range Assay kit (Life Technologies Q10210). Total 2 μg RNAs were used for stranded RNA sequencing library preparation using KCTM Stranded mRNA Library Prep Kit for Illumina (DR08402, Seqhealth, China) following the manufacturer’s instruction. PCR products corresponding to 200–500 bps were enriched, quantified and finally sequenced on Novaseq 6000 sequencer (Illumina) with PE150 model.

### RNA-seq processing and alignment

Raw reads containing more than 2-N bases were first discarded. Then adaptors and low-quality bases were trimmed from raw sequencing reads using FASTX-Toolkit (Version 0.0.13). The short reads less than 16nt were also dropped. After that, clean reads were aligned to the human genome by HISAT2^27^ allowing maximum 4 mismatches. Uniquely mapped reads were used for gene reads number counting and FPKM calculation (fragments per kilobase of transcript per million fragments mapped)^28^.

### Differentially expressed genes (DEG) analysis

The R Bioconductor package DESeq2 (Love, Huber et al. 2014) was utilized to screen out the differentially expressed genes (DEGs). The P value for correction < 0.05 and fold change > 2 or < 0.5 were set as the cut-off criteria for identifying DEGs.

### Alternative splicing analysis

The alternative splicing events (ASEs) and regulated alternative splicing events (RASEs) between the samples were defined and quantified by using the ABLas pipeline as described previously^29^. In brief, ABLas detection of ten types of ASEs was based on the splice junction reads, including exon skipping (ES), alternative 5ʹ splice site (A5SS), alternative 3ʹsplice site (A3SS), mutually exclusive exons (MXE), mutually exclusive 5'UTRs (5pMXE), mutually exclusive 3ʹUTRs (3pMXE), A3SS&ES and A5SS&ES.

To assess KHSRP-regulated ASEs (RASEs), Student’s *t*-test was used to calculate the significance of the ratio alteration of ASEs. ASEs with significant at *P*-value cutoff corresponding to a false discovery rate cutoff of 5% were considered as RASEs.

### RT-qPCR

Glyceraldehyde-3-phosphate dehydrogenase (GAPDH) was used as the control gene for RT-qPCR. The cDNA synthesis was done by standard procedures and RT-qPCR was performed on the Bio-Rad S1000 with Hieff™ qPCR SYBR^®^ Green Master Mix (Low Rox Plus; YEASEN, China). The concentration of each transcript was then normalized to GAPDH mRNA level using 2^−ΔΔCT^ method^30^. Comparison and statistical significance were performed with the two-tail and unpaired Student’s *t*-test. The information of primers for all tested genes was presented in Table S1.

### Functional enrichment and other analysis

The expression analysis of KHSRP in breast cancer patients were performed by UALCAN website^31^. The survival influence of KHSRP expression was assessed by Kaplan–Meier Plotter^32^. Correlation analysis between KHSRP and its regulated DEGs were calculated by GEPIA2^33^. To sort out functional categories of DEGs, Gene Ontology (GO) and KEGG pathways were identified using KOBAS 2.0 server^34^. Hyper geometric test and Benjamini–Hochberg FDR controlling procedure were used to define the enrichment for each pathway.

## Results

### KHSRP had higher expression level and was associated with prognosis in breast cancer patients

**Figure 1:**
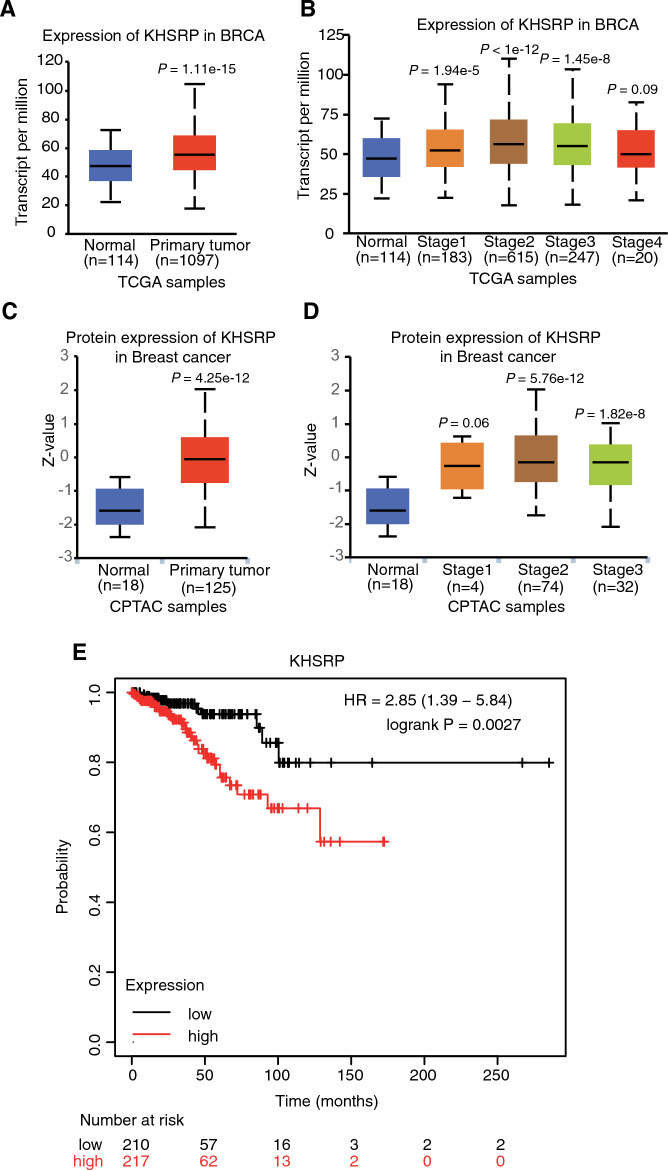
KHSRP expression was significantly increased and associated with the survival time in breast cancer patients. (**A**) Box plot showing the increased expression level of KHSRP in BRCA samples. (**B**) Box plot showing the increased expression level of KHSRP in BRCA samples separated by different stages. The *P*-value was calculated between each tumor stage *vs.* normal samples. (**C**) Box plot showing the increased protein level of KHSRP in breast cancer samples. (**D**) Box plot showing the increased protein level of KHSRP in breast cancer samples separated by different stages. The *P*-value was calculated between each tumor stage *vs.* normal samples. (**E**) Line plot showing the significant association between recurrence free survival time and KHSRP expression in breast cancer patients with low mutation burden.

To obtain a further understanding of KHSRP in breast cancer patients, we utilized online tool UALCAN^31^ to explore its expression pattern in breast invasive carcinoma (BRCA) patients. By classifying TCGA samples into normal and primary tumor groups, we found that KHSRP expression was significantly increased in primary tumor samples (Fig. 1A). By dividing the primary samples into four stages, KHSRP also showed significant higher levels in stages 1–3 compared with normal samples, except stage 4 that had small sample size (Fig. 1B). The protein level of KHSRP was further assessed by using the data from clinical proteomic tumor analysis consortium (CPTAC) group. Similar results were obtained for the protein level of KHSRP in breast cancer patients (Fig. 1C,D), indicating that KHSRP was consistently upregulated in breast cancer at both RNA and protein levels. Finally, we used Kaplan–Meier Plotter^32^ to decipher the association between KHSRP expression and the survival time of breast cancer patients. By setting the survival state as recurrence free survival (RFS) and selecting patients with low mutation burden, we found that higher expression of KHSRP was associated with worse prognosis result (Fig. 1E), indicating that KHSRP expression may be associated with therapeutic efficacy of breast cancer patients. In summary, our results demonstrated the potential oncogenic ability of KHSRP in breast cancer.

### KHSRP knockdown inhibited the pro-tumor features of MDA-MB-231 cells

**Figure 2:**
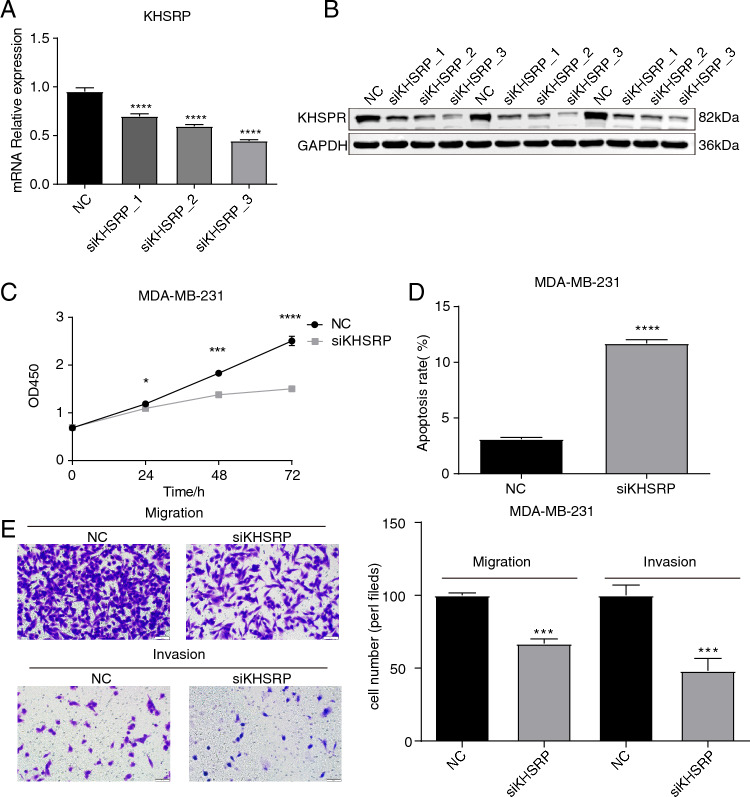
KHSRP knockdown affected cell proliferation, apoptosis, invasion, and migration levels in MDA-MB-231 cells. (**A**) Bar plot showing the RT-qPCR results of control and siKHSRP samples. (**B**) Western blot result showing the decreased expression in siKHSRP samples. (**C**) Line plot showing the cell proliferation result. (**D**) Bar plot showing the cell apoptosis result. (**E**) Cellular staining and bar plot showing the cell invasion and migration results. N = 3; *p-value < 0.05; ***p-value < 0.001; ****p-value < 0.0001; Student’s *t*-test.

Based on the high expression and association with patient’s prognosis of KHSRP in breast tumor^23^, we speculate that KHSRP modulates other phenotypes of tumor cells and regulates expression or other features of downstream targets. Thus, we inhibited KHSRP expression by transfecting small interfering RNA (siRNA) of KHSRP into MDA-MB-231 cell line. All of the three designed siRNAs exhibited significant knockdown efficiency for KHSRP RNA level compared with negative control (NC) by RT-qPCR, and the third siRNA (siKHSRP_3) showed the highest repression level (Fig. 2A). The western blot experiment demonstrated consistent result with that of RT-qPCR, showing the third siRNA most obviously repressed KHSRP protein level (Figs. 2B, S1). We thus chose the third siRNA for following experiments and named it as siKHSRP. We then assessed the phenotype changes of MDA-MB-231 cells by repressing KHSRP. Consistent with previous study^23^, the proliferation rate was significantly repressed and apoptosis level was significantly increased after KHSRP knockdown (Fig. 2C,D). Meanwhile, we calculated the migration an invasion ability of MDA-MB-231 cells. In siKHSRP samples, we detected significantly lower migration and invasion ability compared with NC samples (Fig. 2E). These results together indicate that KHSRP could potentially increase the pro-tumor features of MDA-MB-231 cells, including the metastasis ability.

### Global transcriptome pattern was regulated by siKHSRP in MDA-MB-231 cells

**Figure 3:**
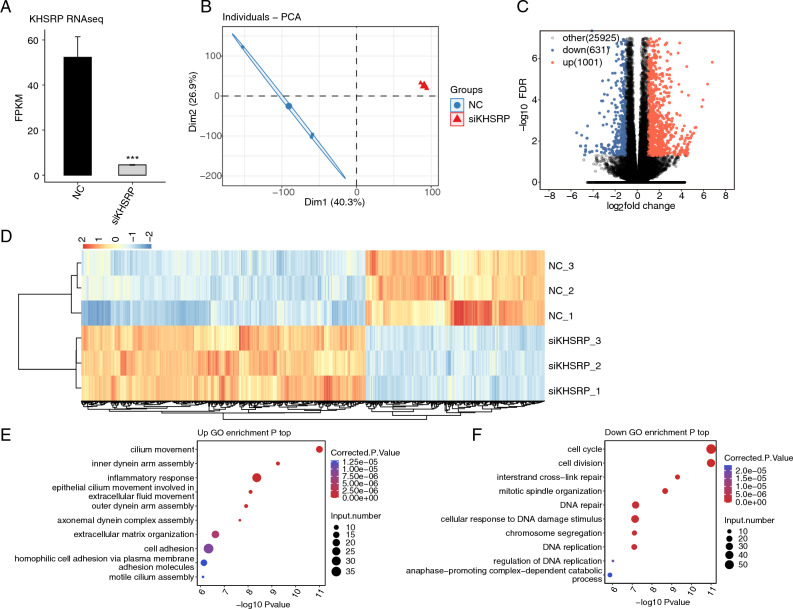
KHSRP globally regulated the transcriptome profile of MDA-MB-231 cells. (**A**) Bar plot showing the expression levels of KHSRP in RNA-seq data. (**B**) PCA base on FPKM value of all detected genes. The ellipse for each group is the confidence ellipse. (**C**) Volcano plot showing all differentially expressed genes (DEGs) between treatment and control samples with DEseq2. FDR < 0.05 and FC (fold change) ≥ 2 or ≤ 0.5. (**D**) Hierarchical clustering heat map showing expression levels of all DEGs (By pheatmap v1.0.12 in R). (**E**) Bubble Diagram exhibiting the most enriched GO pathways of the up-regulated DEGs. (**F**) The same for (**E**) but for the down-regulated DEGs.

To further investigate how KHSRP modulate the pro-tumor features of MDA-MB-231 cells, we performed whole transcriptome sequencing (RNA-seq) experiment for siKHSRP and NC samples. After obtaining the high-quality sequencing reads and aligning them to the human genome, we found the inhibited expression level of KHSRP was also detected in siKHSRP samples from RNA-seq data (Fig. 3A). Principle component analysis (PCA) based on the total expressed genes demonstrated the clear separation between siKHSRP and NC samples (Fig. 3B), indicating that KHSRP knockdown globally changed transcriptome profile. Following differentially expressed genes (DEGs) analysis confirmed the dysregulated transcriptome of MDA-MB-231 cells by identifying 1632 significant DEGs (siKHSRP *vs.* NC), including 1001 up and 631 down DEGs (Fig. 3C). We then plotted the expression pattern of all DEGs using hierarchical clustering heatmap and found the consistent pattern of the three replicates for both siKHSRP and NC groups (Fig. 3D). We also observed that up DEGs in siKHSRP samples were much more than down DEGs, suggesting the potential transcription repression role of KHSRP in MDA-MB-231 cells. To explore the molecular functions of HKSRP downstream targets, we performed GO enrichment analysis for up and down DEGs.

Cell adhesion and extracellular organization associated biological processes, as well as cilium formation and movement, were significantly enriched in up DEGs (Fig. 3E). As tumor cell invasion and migration were tightly associated with cell adhesion and extracellular organization pathways^35^, and cilium assembly associated pathways were associated with tumor development^36^, the enriched pathways for up DEGs could partially explain the decreased invasion and migration ability of MDA-MB-231 cells after KHSRP knockdown. Interestingly, the down DEGs were mainly enriched in cell cycle, DNA damage repair, and cell division associated pathways (Fig. 3F), which could largely elucidate the reason for decreased cell proliferation level in siKHSRP samples. Meanwhile, we also performed KEGG enrichment analysis for both up and down DEGs, and found they showed similar results with the GO analysis (Fig. S2A,B). In summary, the dysregulated genes by siKHSRP showed high consistency with the altered pro-oncogenic features of MDA-MB-231 cells, indicating that KHSRP affects breast cancer cell development by regulating the gene expression pattern.

### Validation of KHSRP-regulated DEGs

**Figure 4:**
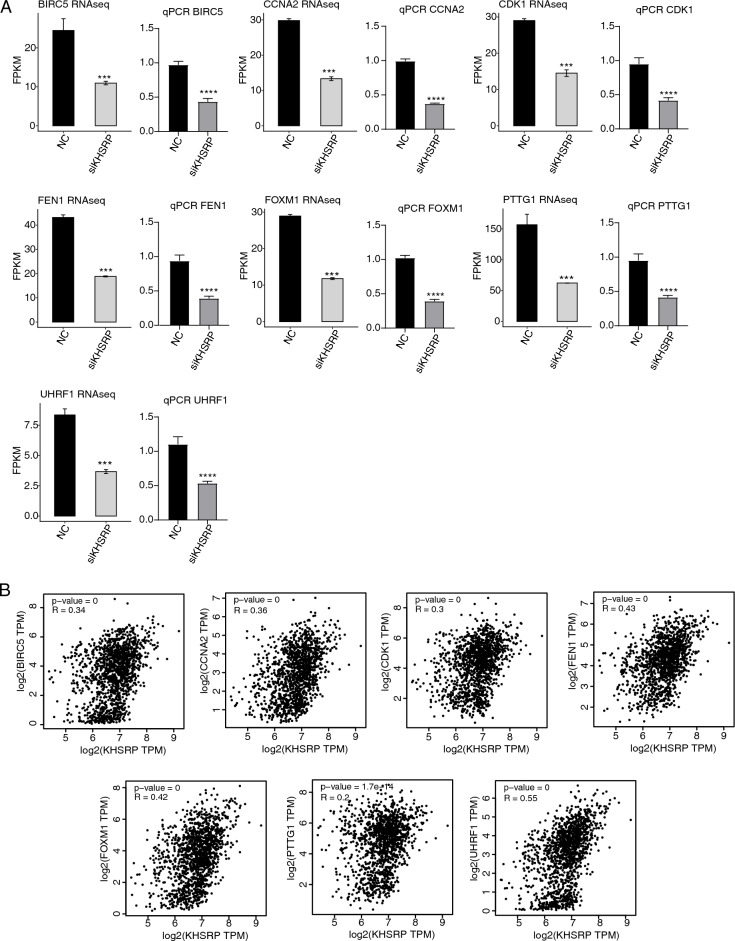
KHSRP regulates the expression of genes in MDA-MB-231 cells. (**A**) Bar plots showing expression level of the selected DEGs using RNA-seq data and RT-qPCR. N = 3; ***p-value < 0.001; ****p-value < 0.0001; Student’s *t*-test. (**B**) Dot plot showing the correlation analysis result between KHSRP and the selected DEGs using BRCA dataset from TCGA.

Based on the above results, we focused our attention on the DNA damage/repair and cell cycle associated genes that were specifically down-regulated by siKHSRP (Fig. 3F). Then we randomly selected 7 genes from these pathways and validated their altered expression level using RT-qPCR method. These 7 genes are *BIRC5* and *CCNA2* from cell cycle pathway, and *CDK1*, *FEN1*, *FOXM1*, *PTTG1*, and *UHRF1* from DNA repair pathway. The RT-qPCR demonstrated that high consistency was identified between the RNA-seq and RT-qPCR results for all selected genes (Fig. 4A). To further confirm the regulation of KHSRP on the DEGs, we performed correlation analysis, and found KHSRP expression was significantly and positively correlated with these genes by GEPIA2 software^33^ using the BRCA dataset from TCGA (Fig. 4B). In summary, we demonstrated that KHSRP has significant influence on the expression of genes from DNA damage/repair and cell cycle pathways.

### KHSRP regulates alternative splicing genes enriched in DNA repair in MDA-MB-231 cells

**Figure 5:**
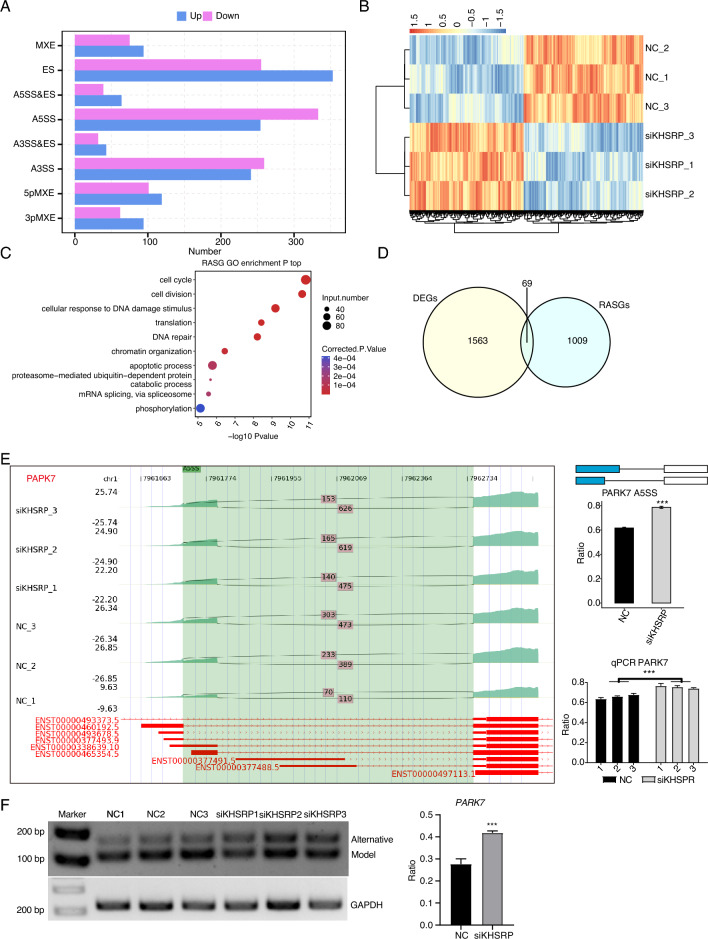
KHSRP regulates alternative splicing genes enriched in DNA repair in MDA-MB-231 cells. (**A**) Bar plot showing the number of all significant regulated alternative splicing events (RASEs). X-axis: RASE number. Y-axis: the different types of AS events. (**B**) Hierarchical clustering heat map showing the values of RASE ratio (by pheatmap v1.0.12 in R). (**C**) Bubble Diagram exhibiting the most enriched GO biological process results of RASGs. (**D**) Venn diagram showing the overlapped gene number of RASGs and DEGs. (**E**) KHSRP regulates alternative splicing of *PAPK7*. Left panel: IGV-sashimi plot showing the regulated alternative splicing events and binding sites across mRNA. Reads distribution of RASE is plotted in the up panel and the transcripts of each gene are shown below. Right panel: The schematic diagrams depict the structures of ASEs. RNA-seq and RT-qPCR validation of RASE were shown at the bottom of the right panel. The ratio was calculated by the alternative spliced reads divided by the sum of alternative spliced and model reads. Error bars represent mean ± SEM. ***p-value < 0.001; Student’s *t*-test. (**F**) RT-PCR showing the significantly regulated AS events between siKHSRP and NC samples. Right panel was the quantitative result. Error bars represent mean ± SEM. ***p-value < 0.001; Student’s *t*-test.

As an RBP, KHSRP has the ability to regulate alternative splicing pattern to modulate the function of target genes. We then investigated how HKSRP affects the AS profile in MDA-MB-231 cells. By using ABLas program that was designed for AS analysis^29^, we identified 1630 HKSRP-regulated AS events (RASEs) with significant difference between siKHSRP and NC (*p*-value < 0.05), indicating the global dysregulation of AS profile by KHSRP knockdown. By classifying these RASEs into different types and included (up) or down (excluded) events, we found ES, A5SS, and A3SS events were the top regulated types (Fig. 5A). We also observed the phenomenon that down RASEs were more than up RASEs, including A5SS and A3SS (Fig. 5A), which is a regulatory feature of KHSRP on AS profile. By plotting the AS ratio of these RASEs, the clear separation between siKHSRP and NC samples was presented with consistent pattern of three replicates for each group (Fig. 5B). After that, functional enrichment analysis of RASE genes (RASGs) showed that cell cycle and DNA damage/repair pathways were among the top enriched pathways (Fig. 5C), which showed high similarity with that of down DEGs (Fig. 3F). Apoptotic process was also enriched in the RASGs (Fig. 5C), associating with the increased apoptotic level in siKHSRP samples. KEGG analysis for RASGs demonstrated that they were enriched in metabolic and several disease pathways (Fig. S3A). We then performed overlapping analysis between RASGs and DEGs, and detected 69 overlapped genes (Fig. 5D), implying that KHSRP could collectively regulate the expression and AS levels of some important genes. To further validate KHSRP regulatory function on AS pattern of DNA damage/repair genes, we designed specific primers that could distinguish model and alternative splicing sites, and performed RT-qPCR experiment for eight selected genes with DNA damage/repair function. The A5SS event of *PARK7* was included in siKHSRP samples and may yield transcript with different 5’UTR (Fig. 5E). The A5SS event of *PARK7* was also confirmed by PCR with two bands that showed higher inclusion of longer transcript in siKHSRP samples (Fig. 5F). The 5pMXE of *UBE2D3* was included in siKHSRP samples and may yield transcript with different 5’UTR (Fig. S3B). The A3SS of *NPM1* was also included in siKHSRP samples and produced more transcripts with shorter coding region (Fig. S3C). For other three RASEs, including ES of *ERCC1* and *CENPX*, and 5pMXE of *UBE2A*, showed significant AS ratio difference that were validated by RT-qPCR and RT-PCR (Fig. S4A–C). A3SS events of *SWI5* and *CSNK1E* were also validated by RT-qPCR and showed high consistency between RNA-seq and RT-qPCR results (Fig. S4D). In summary, these results demonstrated that KHSRP significantly regulated the AS profile of MDA-MB-231 cells, and its AS regulation on DNA damage/repair genes may contribute to its pro-oncogenic functions.

## Discussion

Previous study has demonstrated that KHSRP is highly expressed in breast cancer tissues, and patients with high expression of KHSRP are associated with poor prognosis^23^, but the molecular mechanisms of KHSRP in breast cancer are limited. In this study, we systematically investigated the KHSRP-downstream RNA targets in MDA-MB-231 cells, including changed expression and splicing genes. Besides the higher expression and association with worse RFS of KHSRP in breast cancer patients, we found that KHSRP knockdown could inhibit cell proliferation, migration, and invasion, and promote cell apoptosis. It also demonstrated that KHSRP knockdown could affect the expression and alternative splicing of multiple genes. By analyzing the functional pathways of DEGs and RASGs by siKHSRP, we found that several pathways of down-DEGs and RASGs were related to DNA damage and repair. Therefore, we speculate that KHSRP may play a role in breast cancer by regulating both the expression and alternative splicing of DNA repair functional genes. Meanwhile, other pathways enriched in DEGs and RASGs, could also partially explain the mechanisms of KHSRP pro-oncogenic features. In summary, our study highlighted the potential regulatory mechanisms of KHSRP in breast cancer and identified substantial KHSRP downstream RNA molecules that could be used as potential therapeutic targets for breast cancer in future.

Based on the protein structure of KHSRP, it has the ability to interact with RNAs and regulate their fate in cells, including the well-known RNA decay^37^ and miRNA maturation^38^. In breast cancer, KHSRP was destabilized by the mutant p53–proteasome axis, indicating the functional realization of KHSRP was under p53 mutation pathway^39^. Meanwhile, recent studies also demonstrated the pro-oncogenic function of KHSRP in multiple cancers (see introduction). Based on our results, we speculate that KHSRP fulfils its functions with multiple pathways. By analyzing the DEGs, up DEGs in siKHSRP group were enriched in cilium assembly and movement, extracellular matrix (ECM), and cell adhesion pathways, which could be novel mechanisms in KHSRP-regulated cell migration and invasion ability. The ECM-stimulated cell adhesion has been tightly associated with tumor cell metastasis for a long time^35,40^. Several upregulated genes by siKHSRP from cell adhesion pathway were reported to be involved in the development of breast cancer, including the tumor suppressor PERP^41^, THBS3 that could predict better relapse-free survival^42^, and down-regulated CLDN2 in breast cancer cases^43^. Another interesting discovery was that cilium assembly and movement pathways were distinctly enriched in up DEGs. Primary cilium has been reviewed to influence the cancer hallmarks by involving in multiple important signaling pathways^44,45^. In breast cancer, inhibition of ciliogenesis could promote the tumorigenesis and metastasis^46^, consistent with our finding that upregulated cilium assembly genes coupled with decreased cell migration and invasion abilities. In conclusion, KHSRP knockdown arrested tumor cell metastasis perhaps by promoting the cilium function. Meanwhile, due to the lack of exploration on cilium, the proposed conclusion needs to be further verified with additional experiments.

Another important discovery was that cell cycle and DNA damage/repair genes were deregulated by siKHSRP in both down DEGs and RASGs, suggesting their important roles in repressing proliferation and promoting apoptosis in MDA-MB-231 cells after KHSRP knockdown. The relationship between cell cycle and tumor proliferation is needless to say^47^. For the down DEGs by siKHSRP, cell cycle and division pathways were top enriched, highly consistent with the decreased proliferation level of MDA-MB-231 cells by siKHSRP. We thus focus on discussing the DNA damage/repair pathways and dysregulated AS. DNA damage and following cellular response and repair are essential to preserve the overall genome stability and integrity of cells, the dysregulation of which could lead to uncontrolled biological processes and is the hallmark of cancer^48^. Besides gene expression, previous studies confirmed the important regulatory role of AS in the development of breast cancer^49,50^. Meanwhile, it has been reported that KHSRP regulates AS to involve in multiple tumor types or processes, including resveratrol-inhibited epithelial to mesenchymal transition^51^, and AS dysregulation in lung adenocarcinoma^52^. In this study, we identified the novel downstream targets and molecular functions of KHSRP in breast cancer. Among DNA repair pathway, *PTTG1* and *FOXM1* were significantly downregulated by siKHSRP (Fig. 4). It is reported that PTTG1 and other two genes are associated with endocrine therapy resistance in breast cancer^53^; and PTTG1 was associated with poor prognosis of breast cancer patients and could promote tumor progression by regulating CCNA2 and CCNB2^54^. Transcription factor FOXM1 is a well-studied and oncogenic protein and used as the therapeutic target in breast cancer^55,56^. Interestingly, FOXM1 contributes to doxorubicin resistance of breast cancer cells by regulating DNA repair genes and protecting cancer cells from DNA damage^57^, which is linked to our discovery. We also identified and validated the RASEs of several genes in DNA damage/repair pathways, and identified their functions or dysregulated expression pattern with breast cancer, including *PARK7*^58^, *UBE2A* and *UBE2D3*^59^, *NPM1*^60^, *ERCC1*^61^, *CSNK1E*^62^, *CENPX*^63^, and *SWI5*^64^. These results indicate that KHSRP could simultaneously regulate expression and AS of genes involved in DNA damage/repair pathways. Further experiments are necessary to validate the dysregulated AS events to rule out the false discovery and weak AS events. At the same time, the functions and outcomes of these dysregulated DEGs and RASGs have not been confirmed in breast cancer, and need to be deeply investigated to validate their roles in future studies.

In summary, we have made a systematical analysis for exploring the downstream targets of KHSRP and trying to associate them with the functions of KHSRP in breast cancer cells. Our results indicate that the enriched pathways for both KHSRP-regulated DEGs and AS genes are tightly associated the repressed pro-tumor features in siKHSRP samples, including the cilium assembly and movement, ECM organization, and the cell cycle and DNA damage/repairs pathways emerged in both DEGs and RASGs. We propose that KHSRP could modulate the progression of breast cancer by regulating the expression and AS patterns of these genes, suggesting novel molecular targets and therapeutic methods for breast cancer based on the KHSRP regulatory network in future.

## Supplementary Information

Supplementary Figures.

Supplementary Table S1.

## Data Availability

The raw RNA-seq data has been deposited in the NCBI GEO database with accession ID GSE252239. Meanwhile, as accession number provided in submission system is private, we have created a secure token to allow review of record GSE252239 while it remains in private status: exsbqouwtnovlyj.
