# Author Correction: KHSRP has oncogenic functions and regulates the expression and alternative splicing of DNA repair genes in breast cancer MDA-MB-231 cells

**DOI:** 10.1038/s41598-024-75419-9

**Published:** 2024-10-15

**Authors:** Xuelaiti Paizula, Aliya Wulaying, Dong Chen, Jianghua Ou

**Affiliations:** 1 The Affiliated Tumor Hospital of Xinjiang Medical University, Ürümqi, China; 2 The First Affiliated Hospital of Xinjiang Medical University, Ürümqi, China; 3 Innovation and Research Center, Wuhan Nissi Biotechnology Co., Ltd., Wuhan, China

Correction to: *Scientific Reports* 10.1038/s41598-024-64687-0, published online 26 June 2024

The original version of this Article contained errors in Figure 5, where the sample names in panel (b) and the gene numbers in panel (d) were incorrect. The original Figure 5 and accompanying legend appear below.

**Fig. 5:**
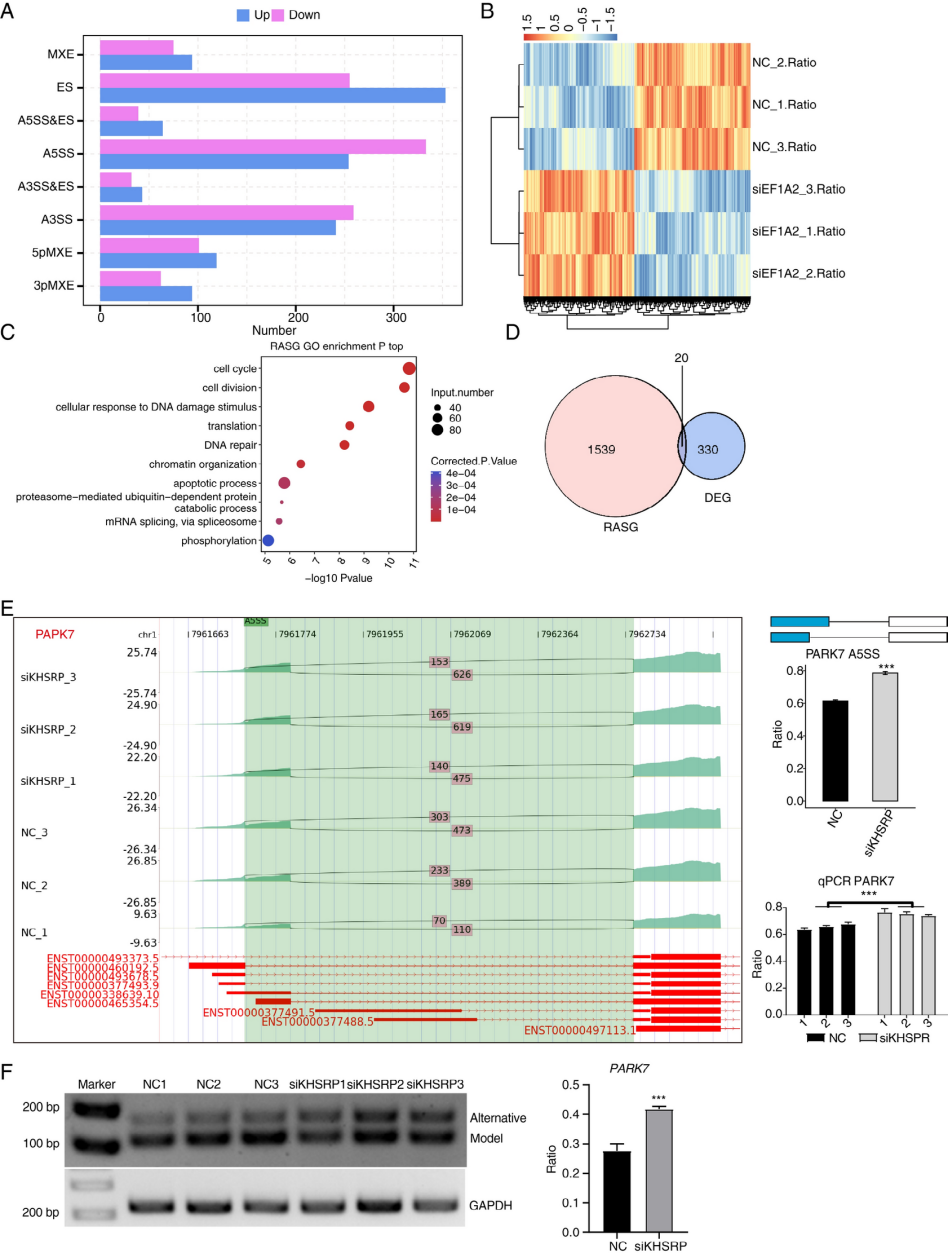
KHSRP regulates alternative splicing genes enriched in DNA repair in MDA-MB-231 cells. (**A**) Bar plot showing the number of all significant regulated alternative splicing events (RASEs). X-axis: RASE number. Y-axis: the different types of AS events. (**B**) Hierarchical clustering heat map showing the values of RASE ratio (by pheatmap v1.0.12 in R). (**C**) Bubble Diagram exhibiting the most enriched GO biological process results of RASGs. (**D**) Venn diagram showing the overlapped gene number of RASGs and DEGs. (**E**) KHSRP regulates alternative splicing of *PAPK7*. Left panel: IGV-sashimi plot showing the regulated alternative splicing events and binding sites across mRNA. Reads distribution of RASE is plotted in the up panel and the transcripts of each gene are shown below. Right panel: The schematic diagrams depict the structures of ASEs. RNA-seq and RT-qPCR validation of RASE were shown at the bottom of the right panel. The ratio was calculated by the alternative spliced reads divided by the sum of alternative spliced and model reads. Error bars represent mean ± SEM. ***p-value < 0.001; Student’s *t*-test. (**F**) RT-PCR showing the significantly regulated AS events between siKHSRP and NC samples. Right panel was the quantitative result. Error bars represent mean ± SEM. ***p-value < 0.001; Student’s *t*-test.

The original Article has been corrected.

