# Supplementary material for: KHSRP has oncogenic functions and regulates the expression and alternative splicing of DNA repair genes in breast cancer MDA-MB-231 cells
Source: Sci Rep. 2024 Jun 26;14:14694. doi: 10.1038/s41598-024-64687-0 (PMC11208542; doi:10.1038/s41598-024-64687-0)
Supplement: Supplementary file 1 — Supplementary Figures. [file 41598_2024_64687_MOESM1_ESM.docx]

**
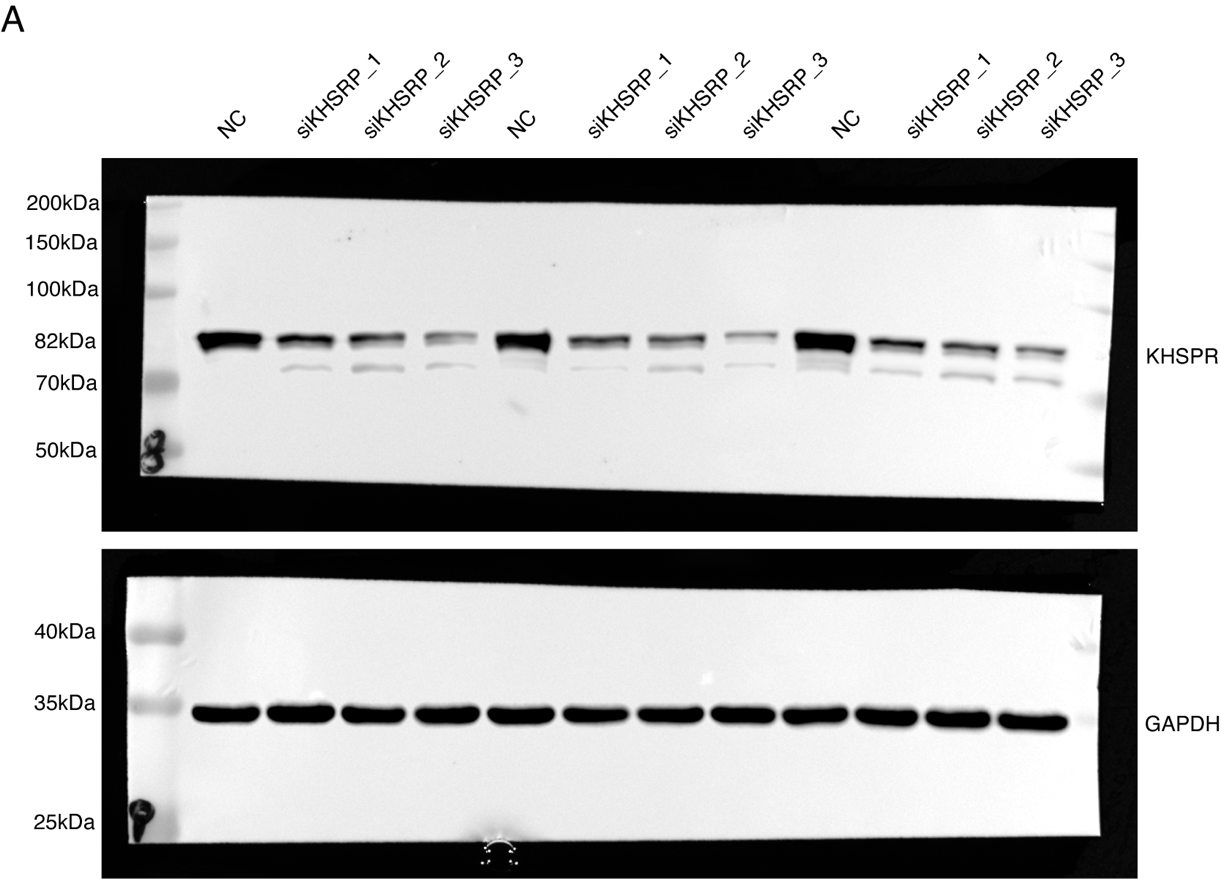
**

**Figure S1. Western blot experiment demonstrated the successful knockdown of KHSRP by siRNAs in MDA-MB-231 cells. The raw gels for western blot were shown.** Due to sample size, we detected two proteins, the target protein and the reference protein, on the same membrane. We cropped the protein positions of the target protein KHSRP and the reference protein GAPDH, and incubated the primary antibodies of the target protein and the reference protein, achieving the goal of detecting the target protein and the reference protein separately. Therefore, the original image we have obtained is a cropped membrane, not a complete whole membrane.

**
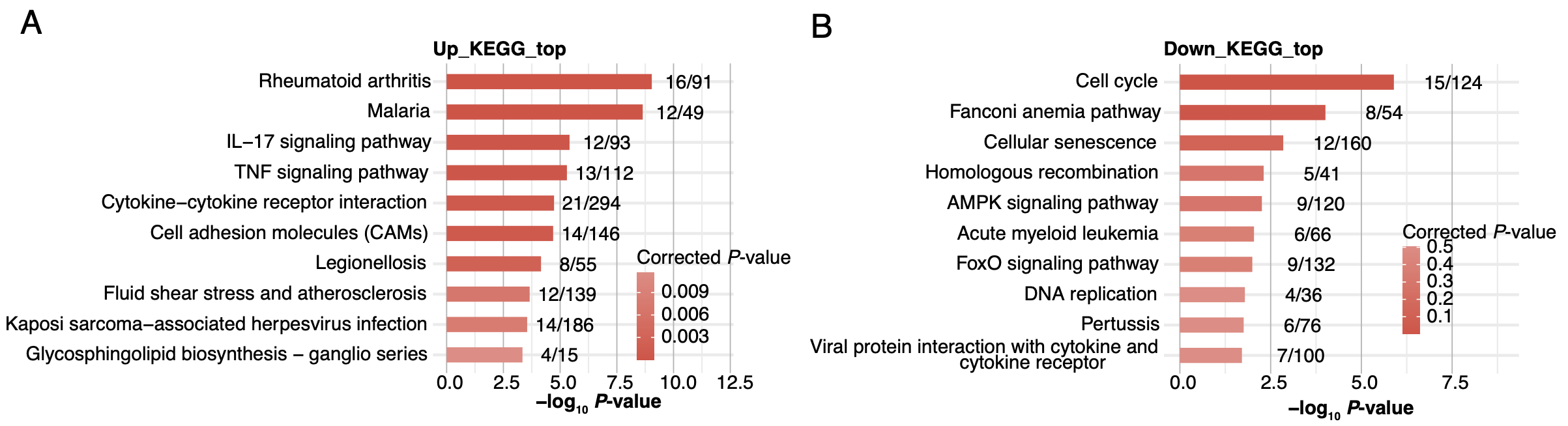
**

**Figure S2. KEGG enrichment analysis for up and down DEGs.**

1. Bar plot showing the top 10 KEGG pathways for up DEGs by siKHSRP.
2. The same as (A) but for the down DEGs.

**
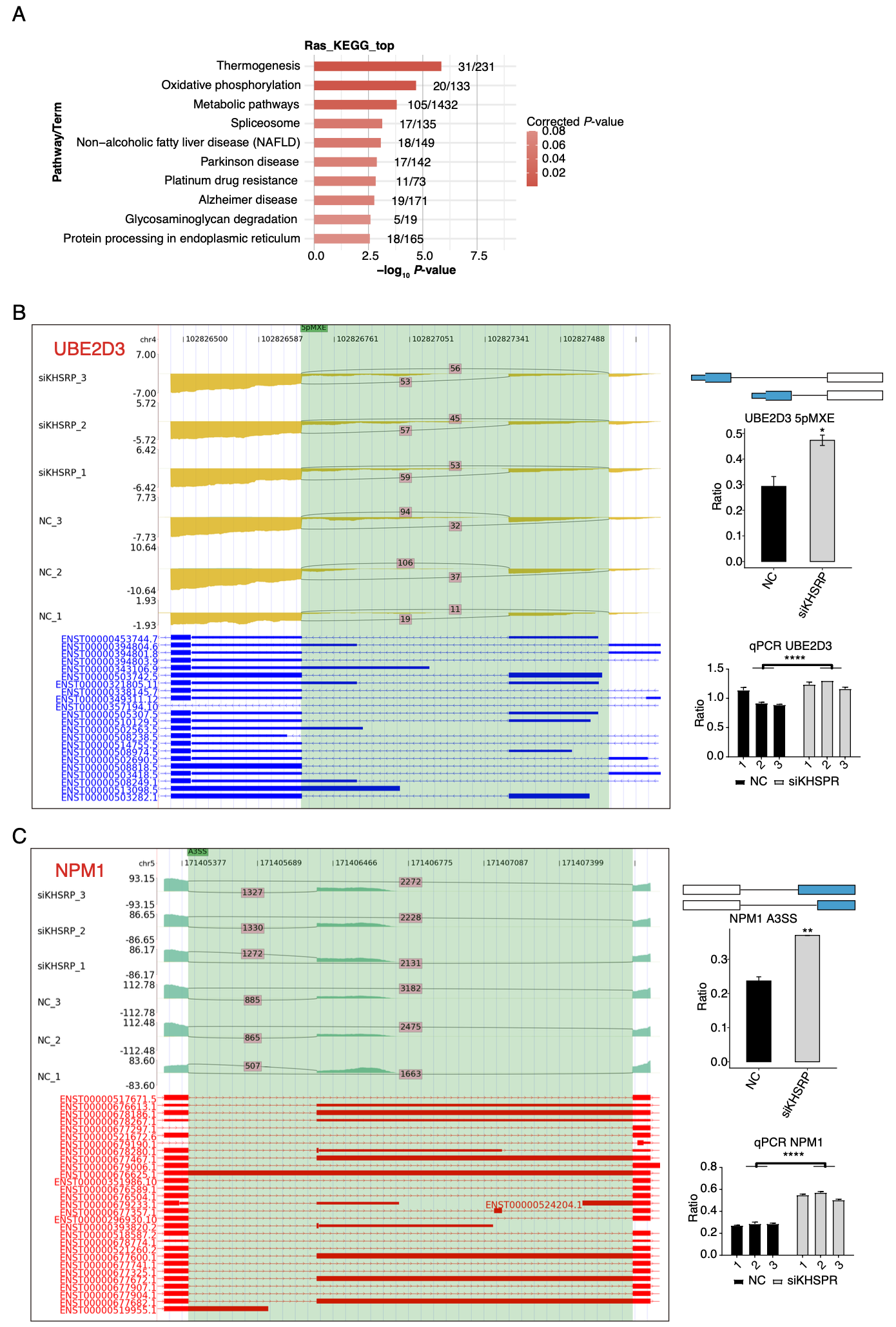
**

**Figure S3. KHSRP regulates alternative splicing in MDA-MB-231 cells.**

1. Bar plot showing the top 10 KEGG pathways for RASGs by siKHSRP.
2. The presentation of 5pMXE RASE of *UBE2D3* that was regulated by KHSRP. Left panel: IGV-sashimi plot showing the regulated alternative splicing events and binding sites across mRNA. Reads distribution of RASE is plotted in the up panel and the transcripts of each gene are shown below. Right panel: The schematic diagrams depict the structures of ASEs. RNA-seq and RT-qPCR validation of RASE were shown at the bottom of the right panel. Error bars represent mean ± SEM. * *p*-value < 0.05, **** *p*-value < 0.0001; Student’s *t*-test.
3. The same as (A) but for the A3SS event of *NPM1*. Error bars represent mean ± SEM. ** *p*-value < 0.01, **** *p*-value < 0.0001; Student’s *t*-test.


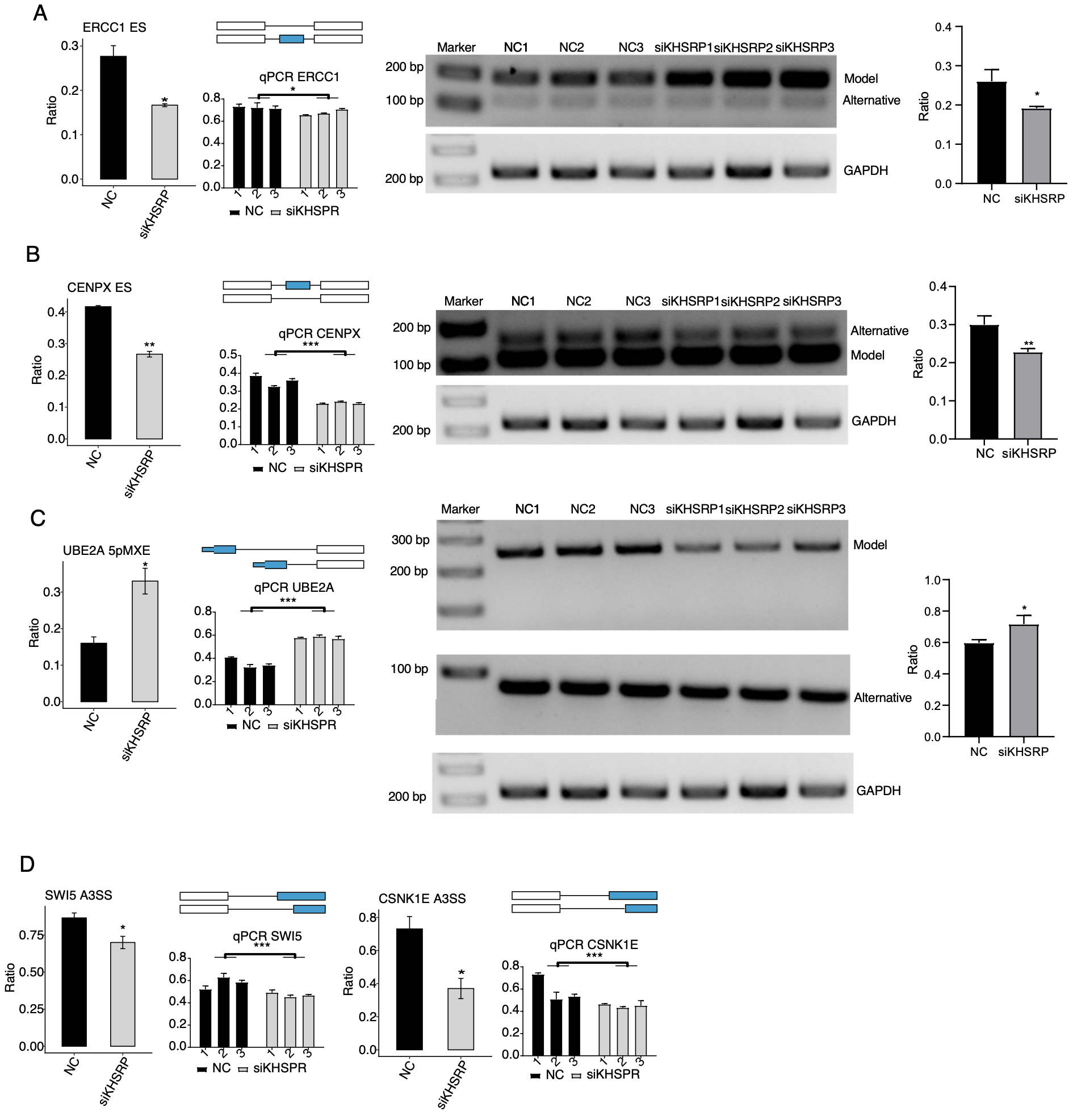


**Figure S4. Bar plot showing the RASEs by siKHSRP and validated results using RT-PCR and RT-qPCR methods.**

1. The ES of ERCC1 was validated by RT-qPCR and RT-PCR experiments. From left to right, the first panel was the RNA-seq result; the second panel was the RT-qPCR result; the third panel was the gel image by RT-PCR; and the last panel was the quantitative result of RT-qPCR.
2. The same as (A) but for the ES of CENPX.
3. The same as (A) but for the 5pMXE of UBE2A.
4. A3SS of SWI5 and A3SS of CSNK1E were shown in this figure. For each RASE, left panel was the RNA-seq result, and right panel was the RT-qPCR result.

Error bars represent mean ± SEM. * *p*-value < 0.05, ** *p*-value < 0.01, **** *p*-value < 0.0001; Student’s *t*-test.
