# Supplementary material for: KHSRP has oncogenic functions and regulates the expression and alternative splicing of DNA repair genes in breast cancer MDA-MB-231 cells
Source: Sci Rep. 2024 Jun 26;14:14694. doi: 10.1038/s41598-024-64687-0 (PMC11208542; doi:10.1038/s41598-024-64687-0)
Supplement: Supplementary file 2 — Supplementary Table S1. [file 41598_2024_64687_MOESM2_ESM.docx]

**Table S1. The sequences table for DEGs and RASEs in the RT-qPCR experiment in this study. F represents forward; R represents reverse; M represents model; and AS represents alternative spliced.**

| BIRC5-F | GAGTGATAGGAAGCGTCTG |
| --- | --- |
| BIRC5-R | GCATCGAGCCAAGTCATT |
| FOXM1-F | CTCGTCAATGCCAGTCTC |
| FOXM1-R | CCCGTGTTTCCAAGTCAG |
| CCNA2-F | CATTAGAGATCCATCTGTTCTG |
| CCNA2-R | TATGAGTAAGACTGGCATCC |
| PTTG1-F | CCTGAAGAGCACCAGATTG |
| PTTG1-R | ATGGTGGAGAGGGCATCT |
| FEN1-F | GTTGGCTGTGTCTGTGTT |
| FEN1-R | CACCATCTTACTCCTCTGAAT |
| CDK1-F | GCAACAGGGAAGAACAGT |
| CDK1-R | TACTCTGACCAAGGCATAAG |
| UHRF1-F | TTGCAGCCTATACCTCAATA |
| UHRF1-R | CTGGACAAAGTACTGGACT |
| PARK7-M-F | CTGGTGTGGGGCTTGTAAAC |
| PARK7-AS-F | CGGGGTGCAGGCTTGTAAAC |
| PARK7-M/AS-R | GATGACCGTCTCCATTTCCT |
| CENPX –M/AS-F | TGCGGAAGCACCTTCTCCAG |
| CENPX -AS-R | TTCGTTGTGGAAGCAGCAGT |
| CENPX -M-R | AAGACCAAAGAAGCAGCAGT |
| ERCC1–M-F | ATTCAGTCACCCGGGAGACGAA |
| ERCC1-AS-F | TGTTCCAGAGACCGGGAGACGAA |
| ERCC1-M/AS-R | TGAGCAGAAACCAGCGGACC |
| CSNK1E–M-F | GGGGCCGCATGAATTTCAGC |
| CSNK1E -AS-F | AGGAAGCCCCGAATTTCAGC |
| CSNK1E -M/AS-R | CAAGCCCGACTACTCTTACCT |
| NPM1–M/AS-F | CACCAAAAGGACCTAGTTCT |
| NPM1-AS-R | TTCAATGCGCTTTTTCTATAC |
| NPM1-M-R | GAGAACCACCTTTTTCTATAC |
| SWI5–M/AS-F | GGACCCTCTTGCGCCATTGA |
| SWI5-AS-R | TGGTTCAGTCCTCCTGAGCC |
| SWI5-M-R | ACTGGATCCATCTCCTGAGCC |
| UBE2D3–M/AS-F | TCGGTGTATGCTCAAAGGTCC |
| UBE2D3-AS-R | CGGTGAAGAGGCGCCTGTGT |
| UBE2D3-M-R | CGGCCCCTCTGCGCCTGTGT |
| UBE2A–M-F | CATCCAAATGTCTATGCAGA |
| UBE2A -AS-F | TTTGAGGATGTCTATGCAGA |
| UBE2A –M/AS-R | TGGATGTTAGAATGGAAGAC |
| GAPDH-F | GGTCGGAGTCAACGGATTTG |
| GAPDH-R | GGAAGATGGTGATGGGATTTC |
